# A modified protocol for the isolation and culture of human umbilical vein endothelial cells

**DOI:** 10.1016/j.bbrep.2025.102221

**Published:** 2025-09-01

**Authors:** Anmol Sandhu, Anannya Parvathi, Jennifer Jane McGhee, Salim Ismail, I-Ping Loh, Bert van der Werf, Jie Zhang, Trevor Sherwin

**Affiliations:** a Department of Ophthalmology, Faculty of Medical and Health Sciences, The University of Auckland, Auckland, New Zealand; b Department of Epidemiology and Biostatistics, Faculty of Medical and Health Sciences, The University of Auckland, Auckland, New Zealand

**Keywords:** Human umbilical vein endothelial cells, HUVECs, Umbilical cord, Vein, Endothelium, Endothelial cells, Cell isolation protocol

## Abstract

The umbilical cord is a valuable source of foetal stem cells, progenitor cells, and early-stage developmental cells, including human umbilical vein endothelial cells (HUVECs). HUVECs are widely used as a model for endothelial biology and are increasingly being investigated for their regenerative potential. Efficient isolation of these cells from the umbilical vein is a critical first step for both research and therapeutic applications. To date, most published protocols utilise Collagenase A for isolation. In this study, we present a modified HUVEC isolation protocol that employs dispase, alongside refined tissue and cell culture handling practices. We characterised the isolated cells using established HUVEC markers CD31 and CD146, and demonstrated in situ detachment of the cells from the vessel wall through immunofluorescence imaging. Our method achieved a success rate exceeding 95.6 % across all umbilical cords processed. These findings highlight the protocol's potential for broad applicability across research settings, using readily accessible reagents and equipment.

## Highlights

• An adapted HUVEC isolation protocol employing dispase for cell detachment.
• Effective cannulation of the umbilical vein using a three-way stopcock.
• Optimization of key HUVEC culture parameters.
• Defined criteria for assessing sample suitability for successful HUVEC isolation.
• Consistent and reproducible results across different umbilical cord samples.

## Graphical abstract

**Figure:**
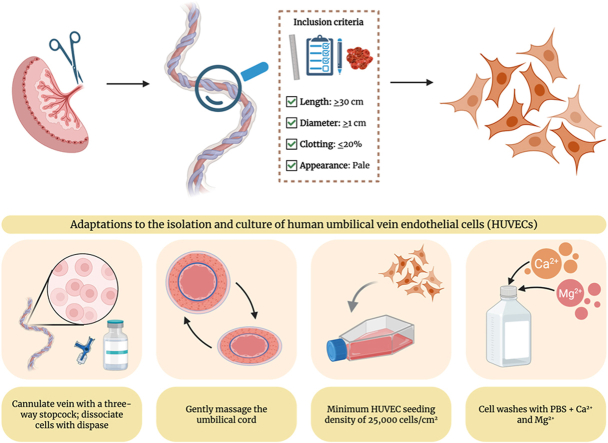

## 1 Introduction

The umbilical cord is a unique structure that connects the foetus to the placenta, serving as a vital conduit for nutrient delivery and waste removal [1]. Structurally, it consists of two arteries that carry deoxygenated, nutrient-depleted blood away from the foetus and a single vein that returns oxygenated, nutrient-rich blood to the foetus [2]. These vessels are embedded in a gelatinous matrix known as Wharton's jelly and enclosed by a simple epithelial layer, referred to as the umbilical cord lining [2]. At term (≥37 weeks of gestation), the average umbilical cord has a length of approximately 50 to60 cm, though normal cord lengths can range from 30 to 100 cm [3]. In addition to its critical role in intrauterine foetal development, the umbilical cord is a rich and readily available source of foetal stem cells, progenitor cells, and early-stage developmental cell types, including mesenchymal stem cells, cord lining epithelial cells, and human umbilical vein endothelial cells (HUVECs) [4,5]. In comparison to adult, embryonic, and induced pluripotent stem cells, cells derived from the umbilical cord are easily sourced from tissue often discarded as medical waste, have high differentiation capabilities, a low risk of teratoma formation, and limited ethical barriers [4]. As such, these cells are easily accessible and ideal candidates for cellular therapeutics and regenerative medicine.

HUVECs, specifically, are sourced from the umbilical vein, which can be distinguished from the two arteries by its larger lumen and thinner vascular wall [2,3]. These cells can be isolated from the umbilical vein through enzymatic digestion and subsequently expanded in culture. Once established, they are widely used as an in vitro model to study various aspects of endothelial cell biology, including responses to hypoxia, inflammation, oxidative stress, and angiogenesis [6,7]. Beyond their utility in basic cell research, HUVECs have also been employed in tissue engineering scaffold development, studies on the anti-inflammatory effects of high-density lipoproteins, investigations into endothelial cell stress responses, and as a model to study cardiovascular disease [6,7]. More recently, their inherent properties as umbilical-derived cells have prompted growing interest in their potential use in therapeutic applications. This includes utilising HUVECs to generate induced pluripotent stem cells and to engineer bone grafts [8,9]. For all such purposes, establishing cultures of HUVECs either by purchasing a cell line or isolating primary cells is an essential first step. Often, cell lines are favoured in place of primary cells due to their convenience, cost-effectiveness, ethical simplicity, and unlimited supply [10]. However, caution is necessary when using cell lines in place of primary cells as long-term culture or genetic manipulation may alter their phenotype and function [10]. As such, cell lines may not entirely represent primary cells, leading to variable results [10]. For certain research applications, particularly those requiring physiological relevance, the use of primary HUVECs is essential. As such, the development of reliable and reproducible protocols for isolating primary HUVECs is crucial to support high-quality, translatable research.

The first attempt to isolate and culture HUVECs was made by Maruyama in 1963, using trypsin treatment at 37***°***C [11]. However, the seemingly over-trypsinisation resulted in detrimental effects on cell viability, with a large quantity of fibroblastic cells dominating cultures, ultimately leading to widespread rejection of these cultures as representative of true endothelial cells [5]. The first bona fide isolation and culture of HUVECs was achieved a decade later by Jaffe et al. who used collagenase, an enzyme that breaks the peptide bonds in collagen, to detach HUVECs from the umbilical vein endothelium [12]. This breakthrough enabled HUVECs to become a widely adopted model for studying both physiological and pathophysiological endothelial processes due to their robust proliferative capacity and relatively low cost [12]. Since then, most published HUVEC isolation protocols use Collagenase A, derived from *Clostridium histolyticum*, typically applied at 37***°***C, with different concentrations and exposure times of collagenase reported between protocols [[13], [14], [15]].

Although collagenase is widely used for HUVEC isolation, it is not without challenges. One practical consideration is that collagenase requires incubation at 37***°***C, necessitating the use of a water bath or incubator, which may introduce a small but notable risk of contamination [5]. Additionally, collagenase activity can vary between batches due to differences in enzymatic composition. As a result, each new batch requires careful calibration of concentration and incubation time, complicating standardisation efforts and contributing to variability across laboratories [5]. This variability is reflected in the literature, where some studies have reported inconsistent results with collagenase use. For example, a study by Taghizadeh et al. (2018) reported that collagenase treatment reduced both cell recovery and marker expression in mesenchymal stem cells isolated from umbilical cord tissue [16]. While the study focused on a different cell type, the shared origin with HUVECs suggests that similar effects could occur. In our experience, collagenase-based HUVEC isolations using 15- and 30-min incubations with 0.2 % Collagenase A resulted in poor cell recovery, and the few HUVECs obtained failed to proliferate. These findings indicate that, under our specific conditions, enzyme choice appeared to influence isolation outcomes.

With this in mind, we investigated alternative enzymes that are commonly used in tissue dissociation protocols, including hyaluronidase and proteases such as trypsin and elastase [[17], [18], [19]]. Trypsin, while frequently used, is known to damage cell membranes and compromise cell viability when used at high concentrations or for prolonged incubation periods [20], as demonstrated in early HUVEC isolation attempts [11]. Among the remaining options is dispase, a neutral protease isolated from *Bacillus polymyxa*, which is reported to be gentle on cell membranes and effective in dissociating cells [21,22]. Given these properties, we evaluated dispase as a potential alternative for isolating HUVECs. In doing so, we also adjusted certain aspects of the isolation process, including vein cannulation technique and umbilical cord handling. The resulting protocol offers an alternative approach that may complement existing protocols.

## 2 Materials and methods

### 2.1 Umbilical cord donors

Umbilical cords were obtained through the Department of Obstetrics and Gynaecology, Faculty of Medical and Health Sciences, at The University of Auckland. Samples were only collected from healthy mothers delivering at term (≥37 weeks of gestation). Informed consent was obtained prior to collection, and samples were de-identified. Within an hour following delivery, the umbilical cord was cut from the placenta, placed into cold Dulbecco's PBS (DPBS) (14190144, Gibco, USA) with 1 % antibiotic-antimycotic (15240-062, Gibco, USA), and stored at 4 °C until use. All umbilical cord samples were processed within 24 h of childbirth. This study was conducted under ethics approval from the New Zealand Health and Disability Ethics Committees. Approval was provided for the project titled ‘Investigating the use of umbilical cord stem cells for the regeneration of adult tissues’. Approval number: 20/NTB/232. Approval date: November 27, 2023.

### 2.2 Fibronectin coating

Culture vessels were coated with fibronectin from human plasma (F2006, Sigma, USA). Fibronectin coating solution was prepared at a concentration of 31.25 μg/mL by dissolving 1 mg of fibronectin in 32 mL of sterile Milli-Q water. This solution can be stored for up to 6 months at 4***°***C. Culture vessels were coated with 0.06 mL of fibronectin coating solution per cm^2^ at ambient temperature for 1 h. After incubation, the solution was removed, and the vessels were washed once with DPBS (14190144, Gibco, USA) [23,24].

### 2.3 Dispase solution

Dispase (17105-041, Gibco, USA) was prepared at a concentration of 0.3 % by dissolving 0.03 g of dispase per 10 mL of DPBS (14190144, Gibco, USA). The solution was syringe filtered using a 0.22 μm filter (SLGVV255F, Millipore, USA), then warmed in a 37***°***C water bath for 30 min prior to use.

### 2.4 HUVEC culture medium

HUVEC culture medium was prepared using Medium 199 (12340-030, Gibco, USA), supplemented with 20 % heat-inactivated foetal calf serum (10091-148, Gibco, USA), 1 % antibiotic antimycotic (15240-062, Gibco, USA), and 1 % GlutaMax (35050-061, Gibco, USA).

### 2.5 Isolation and culture of HUVECs

The umbilical cord was processed under sterile conditions within a tissue culture hood. Its outer surface was washed two to four times with DPBS (14190144, Gibco, USA) until all visible blood was removed. To minimise the risk of contamination from the external environment, approximately 1 cm was removed from each end of the umbilical cord using a scalpel (11–0506, Amtech Medical, New Zealand). Sections of the umbilical cord containing holes or visible clots were also removed. Based on the total length, the umbilical cord was cut into 20 to 30 cm segments for easier handling. Each segment was processed separately, with the resulting cell suspensions pooled at the end.

To isolate HUVECs, the umbilical vein was identified, and a three-way stopcock (394995, Capes Medical, New Zealand) was inserted into one end and secured with elastic (5 to 10 mm width) that had been sterilised in ethanol for 30 min prior to use. Any elastic of similar size may be used for this purpose, provided it can securely hold the stopcock in place. The extension tubing attached to the stopcock was shortened to approximately 1 cm using sterile scissors to allow convenient injection of solutions with a syringe. The umbilical vein was rinsed with DPBS to remove residual blood and reduce red blood cell contamination. Gentle squeezing of the umbilical cord helped dislodge smaller or less visible clots. Washing was repeated until the effluent solution appeared clear. Approximately 1 mL of dispase was then flushed through the vein to displace any remaining DPBS. Subsequently, the opposite end of the umbilical cord was clamped, and the vein was filled with approximately 15 mL of dispase solution (adjusted as needed depending on vein size). The dispase was incubated at ambient temperature for 15 min inside the hood. During this period, the umbilical cord was gently massaged along its length to aid HUVEC detachment. Massaging involved compressing the umbilical cord to approximately 70 % of its width while rubbing each section for a few seconds, covering the entire length of the sample (see Video 1). After incubation, the clamp was removed, and the dispase solution containing detached cells was collected into a 50 mL centrifuge tube. Any remaining HUVECs were recovered by washing the vein four times with 15 mL DPBS. After processing all umbilical cord segments, the dispase and DPBS fractions were centrifuged at 380×*g* for 7 min at ambient temperature. The supernatant was carefully discarded, and the cell pellets were combined together in a single tube and resuspended in HUVEC culture medium.

Supplementary data related to this article can be found online at https://doi.org/10.1016/j.bbrep.2025.102221

The following are the Supplementary data related to this article:

Cells were counted and seeded onto fibronectin-coated culture vessels at a seeding density of approximately 2.5 × 10^4^ cells/cm^2^. The culture medium was changed the day after isolation to remove any remaining red blood cells, and every 2 to 3 days thereafter. HUVEC cultures typically reached confluence within 7 to 9 days.

HUVECs were monitored using brightfield and phase-contrast microscopy (Leica DM IL LED inverted contrasting microscope). Images were captured using a 2.5-megapixel Leica MC120 HD camera on the Leica Application Suite Version 4.4.0.

### 2.6 Subculturing HUVECs

Subculturing of HUVECs was performed at ≥90 % confluence by incubating them with TrypLE™ Express (12604-013, Gibco, USA) for 10 min on a shaker at 37 °C. The cell suspension was centrifuged at 380×*g* for 7 min at ambient temperature. The supernatant was carefully discarded, and the cells resuspended in 1 mL of HUVEC culture medium. A cell count was then performed, and HUVECs were seeded onto new fibronectin-coated culture vessels at a density of approximately 2.5 × 10^4^ cells/cm^2^.

### 2.7 Droplet-digital polymerase chain reaction

Droplet digital PCR (ddPCR) was conducted to measure the levels of HUVEC markers *PECAM1* (platelet and endothelial cell adhesion molecule 1, encoding CD31) and *MCAM* (melanoma cell adhesion molecule, encoding CD146). Cells at three different time points (day 1 of primary culture (P0) and at confluency of P0 and P1) were lysed using TRIzol, and four independent HUVEC samples (n = 4) were collected at each time point. The samples were immediately frozen at -80 °C until RNA extraction. RNA was isolated using the PureLink™ RNA Micro Kit (15596018, Thermo Fisher, USA). RNA yield and quality were then analysed by use of Nanodrop and Agilent TapeStation technologies. A SPUD assay was conducted to assess the presence of PCR inhibitors in the RNA preparations. For samples that passed RNA quality control and the SPUD assay, 240 ng of total RNA was reverse transcribed into cDNA using the SuperScript™ IV VILO™ cDNA synthesis kit (11756050, Thermo Fisher, USA). The synthesis of cDNA was confirmed by gel electrophoresis of β-actin PCR products (PCR amplicon size of 234 base pairs). The expression levels of *PECAM1* (encoding CD31, Hs. PT.58.19487865, Integrated DNA Technologies, USA) and *MCAM* (encoding CD146, Hs. PT.58.40453650, Integrated DNA Technologies, USA) were measured with ddPCR. Seven reference genes were included: *B2M, GUSB, HPRT1, PPIA, RPLP0, TBP, POLR2A*. Droplets were generated using a QX200™ Droplet Generator (Bio-Rad, USA) and PCR was performed in a C100 Touch™ Thermal Cycler (Bio-Rad, USA). The 20 μL reaction mixture contained 10 μL of ddPCR Supermix for Probes with no dUTP (1863024, Bio-Rad, USA), 1 μL of IDT PrimeTime pre-designed gene expression assay, 1 ng of cDNA, and RNase free water. The QX200™ Droplet Reader (Bio-Rad, USA) was used to calculate the number of positive and negative droplets, and thus determine the copies of each gene of interest and reference gene in each sample. The NormFinder plug-in within Microsoft Excel 2021 for Windows (version 16.51) was used to determine the two most stable reference genes, which were found to be *POLR2A* and *PPIA* in this case. The geometric mean of their expression levels in each sample was calculated. The expression level of each gene of interest was then normalised to this geometric mean.

### 2.8 Umbilical cord tissue processing for immunohistochemistry

Before and after HUVEC isolation, different sections of an umbilical cord were washed with DPBS (14190144, Gibco, USA) and frozen in Tissue-Tek Optimal Cutting Temperature compound (4583, Sakura, USA) containing glycols and resins, by immersion in a dry ice/100 % ethanol bath for 2 to 3 min. Frozen tissue was sectioned into 30 μm-thick sections using a cryostat (CryoStar NX50, Thermo Fisher, New Zealand) and collected onto Superfrost® Plus microscope slides (916115, Thermo Fisher, USA). Slides were stored at 4 °C until use. For immunolabelling, slides were washed five times with DPBS without Ca^2+^ and Mg^2+^ (5 min/wash), prior to fixation with 2.5 % paraformaldehyde (P6148-500G, Sigma Merck, USA) for 20 min. This was followed by washing the slides three times with DPBS (10 min/wash).

### 2.9 Cell processing for immunocytochemistry

Some P1 cells were grown on fibronectin-coated coverslips. Upon confluence, the cells were washed once with DPBS containing Ca^2+^ and Mg^2+^ (14040141, Gibco, USA) for 5 min, fixed with 2.5 % paraformaldehyde for 20 min, and washed three times with DPBS without Ca^2+^ and Mg^2+^ (14190144, Gibco, USA) (10 min/wash).

### 2.10 Immunocytochemistry and immunohistochemistry

The expression of CD31 and CD146 was analysed using immunocytochemistry (ICC) and immunohistochemistry (IHC) in four independent HUVEC samples and one umbilical cord tissue sample.

Samples (tissue sections on slides or cells on coverslips in wells) were permeabilised with 100 % methanol (1.06009, Sigma, USA) at -20 °C for 10 min to expose antigens, and then washed three times in DPBS without Ca^2+^ and Mg^2+^ (14190144, Gibco, USA) (10 min/wash). All subsequent washes were conducted using DPBS without Ca^2+^ and Mg^2+^. Samples were blocked to reduce non-specific antibody binding for 1 h in DPBS containing 100 mM glycine (101194 M, VWR Chemicals, United Kingdom), 0.1 % Triton X-100 (9002-93-1, Sigma, USA), and 10 % normal goat serum (16210-072, Gibco, USA), and subsequently washed three times in DPBS with 3 % BSA (ABFF-100G, MP Bio, New Zealand) (10 min/wash). Samples were incubated in the dark overnight at 4 °C with primary antibody in DPBS with 3 % BSA and 0.5 % Triton X-100. Primary antibodies used include: Anti-CD31 (MO823, Dako, Denmark) at a dilution of 1:50 and Anti-CD146 (Ab75769, Abcam, United Kingdom) at a dilution of 1:350.

The following day, samples were washed three times in DPBS with 3 % BSA (10 min/wash), incubated in secondary antibody prepared in DPBS with 3 % BSA for 3 h in the dark at ambient temperature, washed three times in DPBS (10 min/wash), incubated in DAPI (D9542, Sigma, USA, 1:10000 dilution) for 10 min at ambient temperature in the dark, and then washed five times in DPBS (5 min/wash). For CD31 labelling, goat-*anti*-mouse secondary antibody conjugated to Alexa 546 (A-11030, Invitrogen, USA) was used at a dilution of 1:400. For CD146 labelling, goat-*anti*-rabbit secondary antibody conjugated to Cy3 (111-165-003, Jackson ImmunoResearch, USA) was used at a dilution of 1:400. Next, a few drops of CitiFluor anti-fade reagent (17970-25, Electron Microscopy Sciences, USA) were applied to all tissue samples, which were then covered with coverslips and sealed using clear nail varnish. For cell samples, a drop of CitiFluor was applied to a microscope slide, and the coverslip containing the cells was placed on top so that the cells were sandwiched between the slide and coverslip. These were also sealed with clear nail varnish. All slides were stored at 4 °C until microscopic visualisation.

Samples with secondary antibody only were included as experimental controls to ascertain the level of background fluorescence.

### 2.11 Fluorescence microscopy

Fluorescence microscopy was performed using a ZEISS Axio Observer inverted microscope, with images captured using an Axiocam 506 camera with the ZEN 2.6 (Blue edition) software.

### 2.12 Statistical analysis

Data were collated and graphs were generated using Microsoft Excel 2021 for Mac (version 16.51). The logarithm of gene expression was analysed using a linear mixed model. A one-way ANOVA was conducted, followed by priori planned pairwise comparisons between groups. These planned comparisons were specified before data analysis and were based on the estimated parameters and their covariances extracted from the regression output. The analysis was done in R, version 4.0.3 [25], using package Imc4 [26]. Subsequent residual analysis was done using the package DHARMa [27]. Statistical significance was defined as p ≤ 0.05.

## 3 Results

### 3.1 A three-way stopcock secured with elastic is an effective method for umbilical vein cannulation and perfusion

**Fig. 1:**
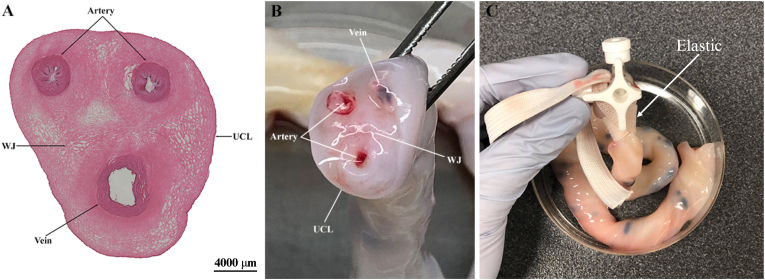
Demonstration of umbilical vein cannulation. (A) Haematoxylin and eosin (H&E)-stained image of a human umbilical cord and (B) Cross-section of the human umbilical cord, showing the two arteries, a vein, Wharton's jelly (WJ), and umbilical cord lining (UCL). The vein has a larger lumen and thinner vascular wall in comparison to the arteries. (C) Umbilical cord vein cannulated with a three-way stopcock, secured in place with sterile elastic.

Cannulating the vein with a three-way stopcock proved the most effective method for passing solutions through the vein, compared to using a cannula or needle (18 gauge). The diameter of the three-way stopcock extension tubing was ideally suited to that of the vein. Securing it in place with elastic proved the most reliable way to prevent fluid loss and displacement (Fig. 1). The elastic could be easily wrapped around the umbilical cord and tightly held the three-way stopcock in position, outperforming other materials trialled, such as surgical thread, cotton twine, cable ties, and string.

### 3.2 Dispase is an effective enzyme for the isolation of HUVECs

**Fig. 2:**
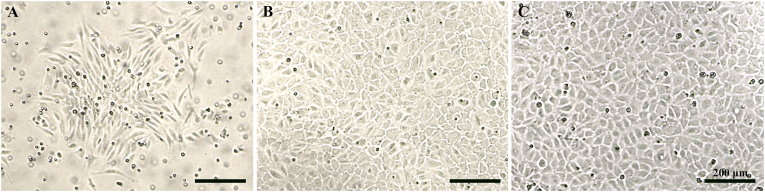
Isolation and culture of HUVECs. Brightfield images showing isolated HUVECs at: (A) 24 h. (B) 7 days, at the point of confluence (P0). (C) 14 days, having reached confluence after subculture (P1). Cells exhibited a cobblestone morphology and formed a monolayer. Scale bar: 200 μm.

Using dispase and a modified isolation procedure, including cannulating the umbilical vein with a three-way stopcock and securing it in place with sterile elastic, HUVECs were successfully isolated and cultured. Across 10 umbilical cords processed (Table 1), an average of 18.0 × 10^5^ cells per umbilical cord was obtained (10.0 × 10^5^ to 27.0 × 10^5^ range). Following isolation, the cells attached in clusters and appeared elongated (Fig. 2A). Once they reached confluence (around day 7 to 9), they exhibited a characteristic cobblestone morphology and formed a monolayer (Fig. 2B). They maintained this morphology following subculture (Fig. 2C).

**Table 1:** Human umbilical vein endothelial cell (HUVEC) yield for ten samples.

Sample number	Length (cm)	Diameter (mm)	Quality	Cell yield
Sample 1	42	10	Suitable	16.0 × 10^5^
Sample 2	44	14	Suitable	25.0 × 10^5^
Sample 3	35	10	Suitable	15.0 × 10^5^
Sample 4	37	9	Suitable	10.0 × 10^5^
Sample 5	75	11	Suitable	18.0 × 10^5^
Sample 6	40	12	Suitable	10.0 × 10^5^
Sample 7	46	11	Suitable	10.0 × 10^5^
Sample 8	45	12	Suitable	24.0 × 10^5^
Sample 9	43	13	Suitable	25.0 × 10^5^
Sample 10	49	13	Suitable	27.0 × 10^5^
**Average**	**45.6**	**11.5**	**Suitable**	**18.0×10^5^**

### 3.3 Best practices for umbilical cord handling and HUVEC culture to enhance cell quality and growth

**Fig. 3:**
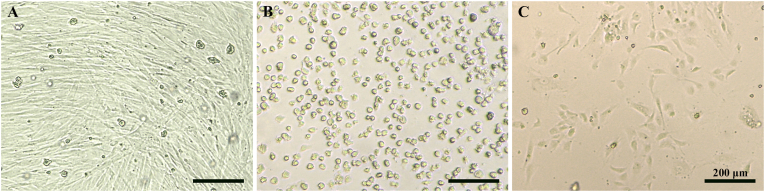
Impact of smooth muscle cell contamination, calcium depletion, and low seeding density on HUVEC growth. Brightfield images showing: (A) Isolation of smooth muscle cells due to vigorous handling of the umbilical cord. (B) Changes in HUVEC morphology and cell detachment following washing with DPBS without Ca^2+^ and Mg^2+^. (C) Growth of HUVECs seeded at a low seeding density of 2.0 × 10^4^ cells/cm^2^; cells adopted a fibroblastic morphology and failed to proliferate. Scale bar: 200 μm.
(2) HUVECs changed morphology and detached when washed with DPBS without Ca^2+^ and Mg^2+^ (Fig. 3B). As such, DPBS containing Ca^2+^ and Mg^2+^ must be used when washing cells.
(3) HUVECs failed to proliferate when seeded at low seeding densities. A minimum seeding density of 2.5 × 10^4^ cells/cm^2^ was required for cells to reach confluence. When seeded below this density, cells adopted a fibroblastic morphology (Fig. 3C). Following isolation, cells from one umbilical cord were typically seeded onto 3 to 5 T-25 flasks (depending on the HUVEC yield) to achieve this minimum seeding density.

When processing umbilical cords, the following observations were made regarding optimal handling of samples and cell cultures:

(1) During dispase incubation, it is important to massage the umbilical cord gently, as more vigorous handling led to the isolation of smooth muscle cells, which subsequently dominated cultures (Fig. 3A). Gentle massaging can be achieved by applying slight pressure to the umbilical cord (compressing it to approximately 70 % of its original width) rather than squeezing it entirely (see Video 1). In instances where smooth muscle cells are isolated, cultures should be discarded.

### 3.4 Umbilical cords exhibit inter-sample variation

**Fig. 4:**
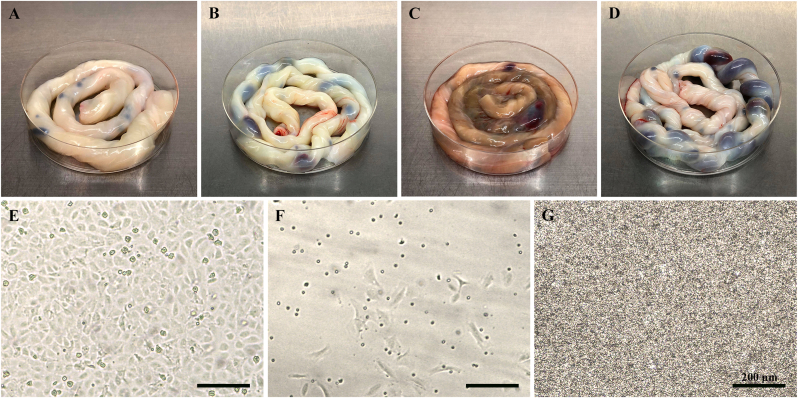
Influence of umbilical cord variability on HUVEC yield and quality. (A) Umbilical cord with no clots, a length of 45 cm, and a diameter of 18 mm. (B) Umbilical cord with a length of 62 cm and a diameter of 8 mm. (C) A discoloured and bruised umbilical cord. (D) Umbilical cord containing several blood clots. (E) Brightfield image of HUVECs isolated from sample A, showing good cell yield and morphology. (F) Brightfield image of HUVECs isolated from sample C, showing low cell yield and fibroblastic morphology. (G) Brightfield image of HUVECs isolated from sample D, showing red blood cell contamination. Scale bar: 200 μm.

During this study, 45 umbilical cords were processed, revealing significant variation in length, diameter, clotting, and overall appearance. It was initially thought that umbilical cords of greater length would give higher cell yields, which was true for some samples. For instance, a 75 cm umbilical cord produced a cell yield of 18.2 × 10^5^ cells, while a 35 cm umbilical cord yielded 5.2 × 10^5^ cells. However, often shorter umbilical cords with larger diameters provided higher cell yields. For example, a 45 cm umbilical cord with a diameter of 20 mm yielded 16.9 × 10^5^ cells (Fig. 4A), whereas a 65 cm umbilical cord with a diameter of 8 mm yielded 12.35 × 10^5^ cells (Fig. 4B). Umbilical cords that appeared bruised or discoloured (Fig. 4C), likely due to damage during or after childbirth, typically resulted in poor-quality cell isolates, with many cells adopting a fibroblastic morphology (Fig. 4F). In contrast, cells from pale-coloured umbilical cords produced higher-quality isolates (Fig. 4E). Significant clotting in the vein posed additional challenges by obstructing the passage of solutions through the vein (Fig. 4D), necessitating the removal of affected sections. Clots could sometimes be cleared by passing DPBS through the vein. However, residual blood often remained and was dislodged after dispase incubation, leading to significant red blood cell contamination of cultures (Fig. 4G).

Overall, this protocol resulted in a culture success rate exceeding 95.6 % (43 out of 45 isolations produced isolates that reached confluence). In cases where cell yields were insufficient, the umbilical cord samples were found to be suboptimal, often exhibiting discolouration, excessive clotting, or inadequate size. Based on these findings, we recommend processing umbilical cords that meet the following criteria for optimal cell yields: a length of at least 30 cm, a diameter of at least 10 mm, less than 20 % blood clotting along the length, and a pale appearance.

### 3.5 Isolated HUVECs express *PECAM1* (CD31) and *MCAM* (CD146)

**Fig. 5:**
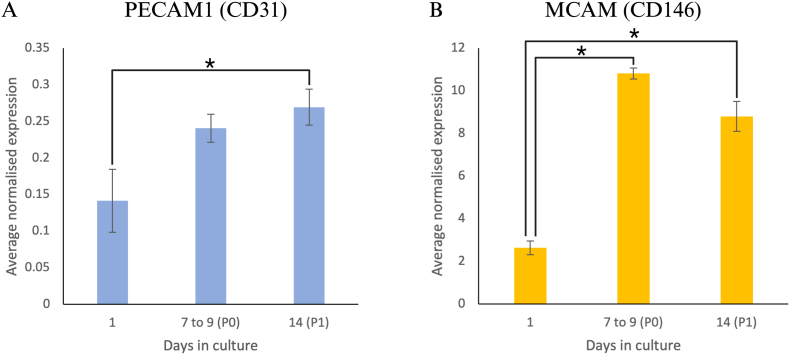
Expression levels of CD31 and CD146 in cultured HUVECs. Average normalised gene expression of HUVEC markers (A) *PECAM1* (CD31) and (B) *MCAM* (CD146) in HUVECs isolated from four different umbilical cords (n = 4). HUVECs expressed *PECAM1* (CD31) and *MCAM* (CD146) on day 1 following isolation, at confluence (day 7 to 9, P0), and following subculture on day 14 (P1). Overall, the expression of both markers increased from day 1 to 14. Error bars represent the standard error of the mean. Asterisks indicate statistical significance (*p* ≤ *0.05).*

Isolated HUVECs exhibited consistent gene expression of *PECAM1* (CD31) and *MCAM* (CD146), both established HUVEC markers, on day 1 post-isolation, day 7 to 9 (P0) when cells reached confluence, and day 14 (P1) following subculture. Across the three timepoints, *PECAM1* (CD31) showed average normalised gene expression between 0.14 and 0.27, while *MCAM* (CD146) showed average normalised gene expression between 2.63 and 10.90 (Fig. 5), demonstrating that isolated cells consistently express CD31 and CD146 during culture.

### 3.6 Isolated HUVECs show localised expression of CD31 and CD146

**Fig. 6:**
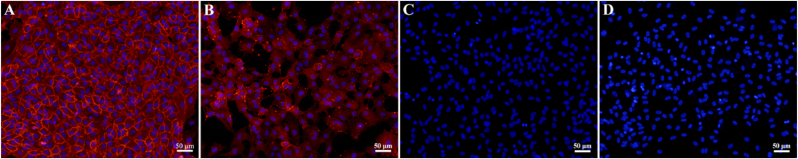
Detection of CD31 and CD146 protein expression in cultured HUVECs labelled with (A) CD31 (red) and (B) CD146 (red). Cell nuclei were counterstained with DAPI (blue). Secondary antibody-only controls (C and D). Scale bar: 50 μm.

HUVECs displayed strong CD31 expression on the cell membrane, with additional cytoplasmic labelling observed, highlighting their characteristic cobblestone morphology (Fig. 6A). CD146 expression was present on the cell membranes and in the cytoplasm, although it was less distinct than the prominent CD31 labelling (Fig. 6B).

### 3.7 CD31^+^ cells are detached from the umbilical vein endothelium during the isolation process

**Fig. 7:**
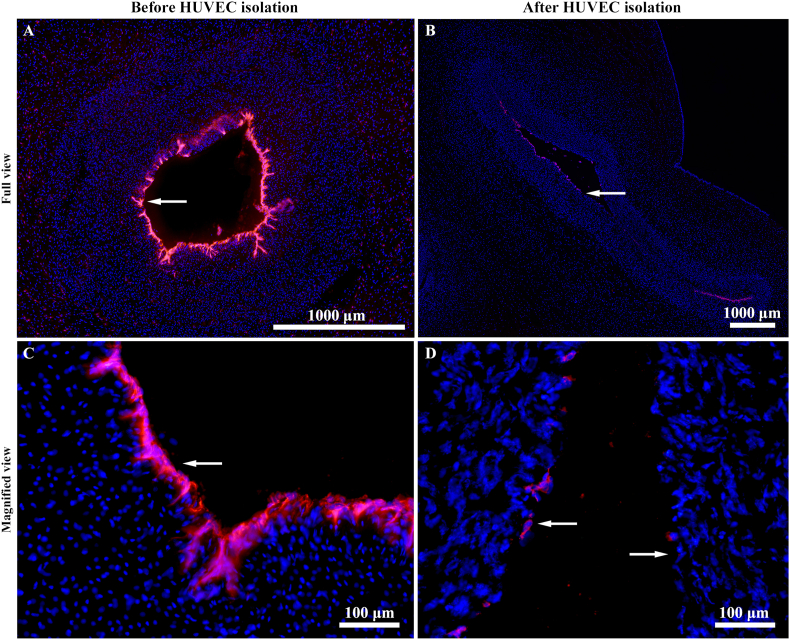
In situ detachment of HUVECs from the umbilical vein. Fluorescence images of the umbilical vein immunolabelled with CD31 (red) in panels (A and C) before, and (B and D) after, HUVEC isolation. HUVECs were present along the entire lining of the umbilical vein (indicated by arrows) prior to isolation, whereas, following isolation only a small proportion of these cells remained. Cell nuclei were labelled with DAPI (blue). Scale bars: (A and B) 1000 μm; (C and D) 100 μm.

To confirm that the correct cell type had been isolated, different portions of the same umbilical cord were sectioned and labelled for CD31 expression, both before and after HUVEC isolation. As previously mentioned, CD31 serves as a gold-standard marker for identifying HUVECs. Before isolation, CD31^+^ cells were present along the entire lining of the umbilical vein vascular wall (Fig. 7A and C). In contrast, after isolation, only a small population of these cells remained (Fig. 7B and D), indicating that a substantial proportion had been successfully isolated.

## 4 Discussion

HUVECs are widely used as an in vitro model for studying endothelial cell processes and have more recently attracted interest for their potential therapeutic applications [[7], [8], [9]]. For all such purposes, successful HUVEC isolation is a critical first step. Using this modified protocol, we successfully isolated HUVECs under our experimental conditions. In culture, the cells exhibited a characteristic cobblestone morphology, typical of HUVECs [5]. Immunolabelling confirmed membranous and cytoplasmic expression of CD31 and CD146, both considered gold-standard markers for HUVEC identification. This was further supported by gene expression of *PECAM1* (CD31) and *MCAM* (CD146). To further validate that the isolated cells were indeed HUVECs, CD31 immunolabelling of an umbilical vein section before and after isolation revealed that many of the resident cells had successfully been removed from the vein. Together, these findings confirm that the isolated cells were HUVECs and that they maintained their phenotype and characteristic features in culture.

Defining features of this modified HUVEC isolation protocol include the use of dispase for cell dissociation, vein cannulation using a three-way stopcock secured with sterile elastic, and cell seeding at a minimum density of 2.5 × 10^4^ cells/cm^2^. Dispase, a neutral protease isolated from *Bacillus polymyxa*, is known to be an effective and gentle enzyme [21,22], making it particularly suitable for isolating primary endothelial cells. In comparison, many existing published protocols (Table 2) use collagenase for dissociation [[13], [14], [15]], while others use trypsin [5,11]. Moreover, many protocols (Table 2) employ a cannula, vinyl tube, or needle secured with string, cable ties, or a clamp to perfuse the umbilical vein [5,11,[13], [14], [15]]. However, in our trials, this method resulted in significant fluid leakage and subsequent cell loss due to a mismatch between the diameter of the standard cannula and the typically larger diameter of the umbilical vein. Additionally, surgical thread proved difficult to use effectively, as it lacks the strength and grip needed to wrap securely around the thick umbilical cord and hold the cannula in place. In contrast, the use of a three-way stopcock was found to be more effective. The 3 mm diameter of the extension tubing is well matched to the size of the umbilical vein, minimising leakage. Once inserted, the stopcock can be reliably secured using sterile elastic, which offers the necessary flexibility and tension to hold it firmly in place. This modification not only reduces cell loss but also improves the overall efficiency of the isolation process. In future, studies that directly compare different isolation methods under standardised conditions are needed to better evaluate their relative effectiveness.

**Table 2:** Summary of dissociation enzymes and vein cannulation techniques used in published human umbilical vein endothelial cell (HUVEC) isolation protocols.

Reference	Title	Dissociation enzyme	Vein cannulation technique
[5]	A new, rapid and reproducible method to obtain high quality endothelium in vitro	Trypsin-EDTA	Cannula secured with cable ties
[11]	The human endothelial cell in tissue culture	Trypsin	Vinyl tube
[13]	A protocol for isolation and culture of human umbilical vein endothelial cells	Collagenase	Cannula secured with string
[14]	Isolation of human umbilical vein endothelial cells	Collagenase	Needle secured with a clamp
[15]	Endothelial cell culture: protocol to obtain and cultivate human umbilical endothelial cells	Collagenase	Needle secured with string

Obtaining a high yield of HUVECs is essential for both experimental and therapeutic applications, to allow for sufficient cell numbers for downstream analysis or treatment. Jimenez et al. [5] proposed that HUVEC isolation efficiency can be calculated by relating the surface area of the umbilical vein to the expected number of HUVECs. Given that the average diameter of the vein is 2.7 mm, a 10 cm long umbilical cord segment will contain approximately 8.5 cm^2^ of endothelial vein surface. In vivo, a HUVEC has a reported diameter of 15 μm, covering a surface area of approximately 180 μm^2^ [5]. Based on these values, every centimetre of umbilical cord tissue should house approximately 4.5 × 10^5^ cells. According to these calculations, Jimenez et al. [5] predicted the efficiency of HUVEC isolation to be between 3 and 11 % at best. Our calculated isolation efficiency is between 6 and 17 %, thus comparable to the published literature.

While a high HUVEC recovery is desirable, achieving 100 % recovery from the umbilical vein is not feasible with current methods. Prolonged dispase incubation, which could potentially release additional HUVECs, also risks digesting deeper layers of the vessel wall and liberating smooth muscle cells. This highlights a key limitation of HUVEC isolation protocols: there is no definitive method to extract all resident HUVECs without also compromising the purity of the population by releasing non-HUVEC cell types. As such, a careful balance must be maintained between effective HUVEC isolation and preserving vascular wall integrity. It is preferable to leave a portion of the endothelial layer intact rather than risk contamination with non-endothelial cells. This compromise is evident in our CD31 labelling of the umbilical vein after isolation, where some CD31^+^ cells remained, indicating that controlled digestion successfully prioritised purity over total recovery.

Furthermore, significant variability was observed between umbilical cord samples in terms of length, diameter, degree of clotting, and overall appearance. Among these factors, cord length and diameter primarily influenced the quantity of cells isolated, while the degree of clotting and discolouration impacted cell quality. Based on our observations, we recommend excluding umbilical cords that are markedly discoloured or exhibit more than 20 % clotting along their length, as these are more likely to yield contaminated isolates containing fibroblasts and red blood cells. The length and diameter of the umbilical cord processed can be adjusted based on the desired cell yield; however, as a general guideline, we propose using umbilical cords with a minimum length of 30 cm and a diameter of at least 10 mm to ensure adequate cell recovery. Overall, these findings can inform the development of inclusion and exclusion criteria for selecting suitable umbilical cords, thereby improving the consistency and quality of HUVEC isolations for research and therapeutic applications.

## CRediT authorship contribution statement

**Anmol Sandhu:** Writing – original draft, Methodology, Investigation, Formal analysis, Data curation. **Anannya Parvathi:** Project administration. **Jennifer Jane McGhee:** Project administration. **Salim Ismail:** Project administration. **I-Ping Loh:** Project administration. **Bert van der Werf:** Formal analysis. **Jie Zhang:** Writing – review & editing, Supervision, Funding acquisition. **Trevor Sherwin:** Writing – review & editing, Supervision, Funding acquisition.

## Funding sources

This work was supported by funding from The Save Sight Society of New Zealand and The Lottery Health Commission.

## Declaration of competing interest

The authors declare that they have no known competing financial interests or personal relationships that could have appeared to influence the work reported in this paper.

## Data Availability

Data will be made available on request.
